# Supplementary material for: A Global Screen for Assembly State Changes of the Mitotic Proteome by SEC-SWATH-MS
Source: Cell Syst. 2020 Feb 26;10(2):133–155.e6. doi: 10.1016/j.cels.2020.01.001 (PMC7042714; doi:10.1016/j.cels.2020.01.001)

Q9Y485 | DMXL1_HUMAN | DMXL1 XL1
Monomer MW [kDa]: 337.839 Monomer expected elution fraction: 30

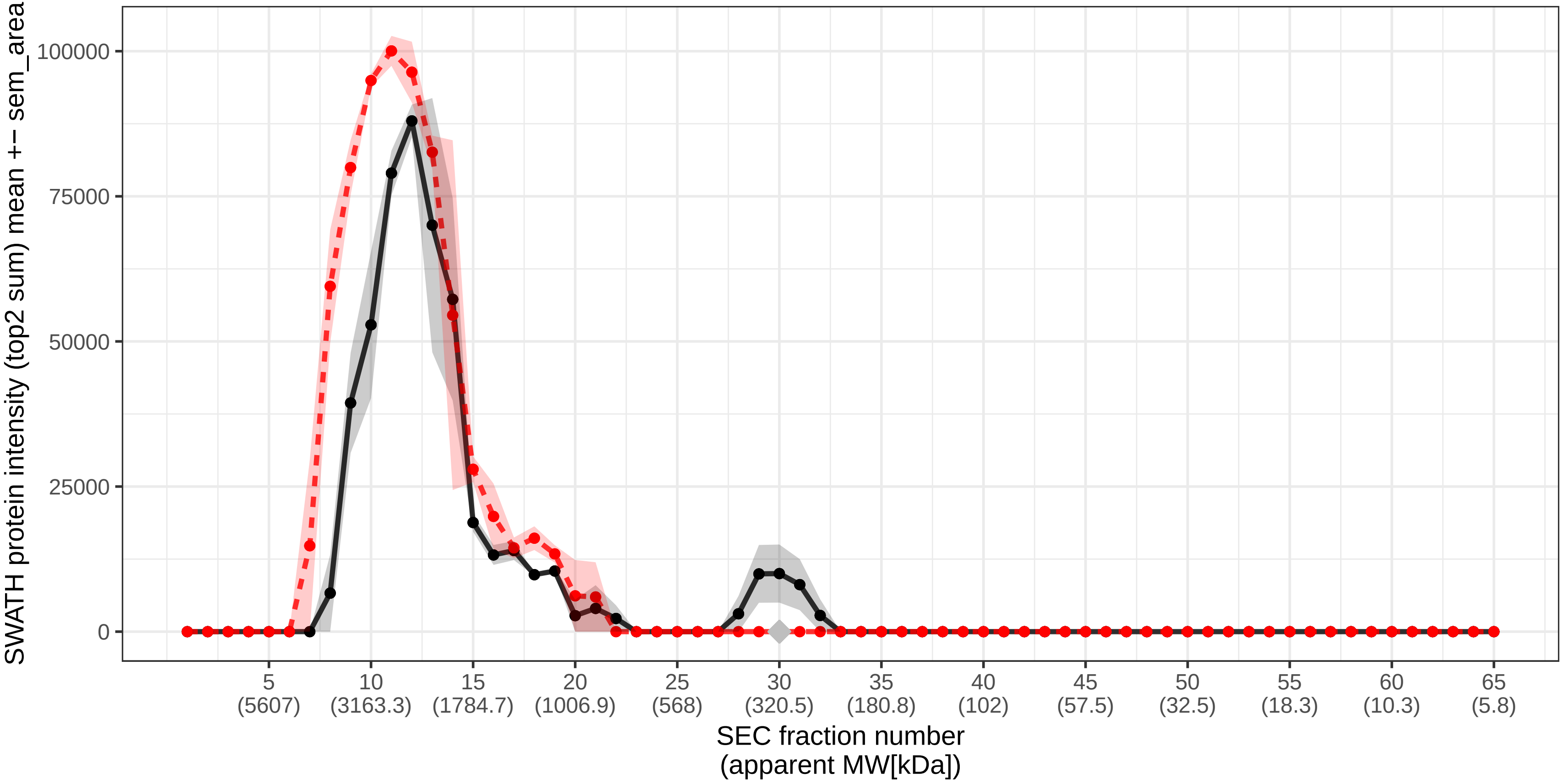

Supplement: Data S1. SEC-SWATH-MS Protein Chromatograms, Related to Figure 1 [file mmc6.zip › SECchrom_Q9Y485_DMXL1_HUMAN_DMXL1_XL1.pdf]
